# Risk Stratification Using Mitral INsufficiency Echocardiographic Score 2 in Dogs With Preclinical Mitral Valve Disease

**DOI:** 10.1111/jvim.70215

**Published:** 2025-08-27

**Authors:** Tommaso Vezzosi, Giovanni Grosso, Liva Vatne, Francesco Porciello, Elena Dall'Aglio, Carlo Guglielmini, Helena Broch, Dave Dickson, Marta Croce, Valentina Patata, Federica Marchesotti, Rosalba Tognetti, Mark Rishniw, Oriol Domenech

**Affiliations:** ^1^ Department of Veterinary Sciences University of Pisa Pisa Italy; ^2^ Anicura Oslo Animal Hospital Oslo Norway; ^3^ Department of Veterinary Medicine University of Perugia Perugia Italy; ^4^ Clinica Veterinaria Milano Sud Milano Italy; ^5^ Department of Animal Medicine Production and Health, University of Padua Padova Italy; ^6^ Anicura Clinica Veterinaria CMV Varese Varese Italy; ^7^ HeartVets UK; ^8^ Anicura Istituto Veterinario Novara Novara Italy; ^9^ College of Veterinary Medicine, Cornell University Ithaca New York USA

**Keywords:** asymptomatic, cardiology, mitral regurgitation, prognosis, survival

## Abstract

**Background:**

Outcome‐based cardiac risk stratification schemes are lacking for preclinical myxomatous mitral valve disease (MMVD). The Mitral INsufficiency Echocardiographic (MINE) score was developed as an easy‐to‐use severity classification of MMVD.

**Hypothesis/Objectives:**

The primary aim was to verify the efficacy of the MINE score in stratifying the cardiac risk in preclinical MMVD. Secondary aims were to evaluate a simplification of the original score and propose a definition of “advanced B2”.

**Animals:**

Seven hundred forty‐nine dogs with preclinical MMVD.

**Methods:**

Retrospective, multicenter, cohort study. Clinical usefulness of the MINE score was tested by evaluating its association with median time to cardiac event. The Cox proportional hazards regression was used to evaluate the echocardiographic independent predictors of cardiac endpoint. Long‐term outcome was analyzed using Kaplan–Meier curves and log‐rank test.

**Results:**

Based on multivariate analysis, a simplified version of the MINE score was redefined including only the left atrium‐to‐aorta ratio, the left ventricular end‐diastolic diameter, and the E‐wave velocity. Mild cases had longer median time to cardiac event [2604 days, 95% confidence interval (CI) 2344–2604 days] in comparison to moderate (1216 days, 95% CI 998–1882 days) and severe cases (718 days, 95% CI 599–980 days; *p* < 0.001). Among stage B2, severe cases had shorter median time to cardiac event (718 days, 95% CI 599–980 days) in comparison to moderate (1141 days, 95% CI 980–1725 days) and mild cases (not available; *p* < 0.001).

**Conclusions and Clinical Importance:**

For this study cohort, the simplified version of the MINE score was clinically effective for risk stratification of preclinical MMVD. Dogs in stage B2 classified as “severe” can be defined “advanced” B2.

**Abbreviations**
CHF: congestive heart failure
CI: confidence interval
E‐vel: E‐wave transmitral peak velocity
FS: fractional shortening
LA/Ao: left atrium‐to‐aorta ratio
LVIDDn: left ventricular end‐diastolic diameter normalized for body weight
MINE: Mitral INsufficiency Echocardiographic
MMVD: myxomatous mitral valve disease

## 1 Introduction

Myxomatous mitral valve disease (MMVD) is the most common acquired cardiac disease in dogs [1, 2]. Most dogs with MMVD remain asymptomatic for years, and approximately one‐third experience cardiac death or are euthanized because of progressive heart failure [3].

Current ACVIM consensus guidelines suggest classifying dogs with preclinical MMVD into stage B1 and B2 based on clinical, radiographic, and echocardiographic findings [4]. It is noted that while the prognosis for dogs in stage B1 is generally favorable [3, 5], recent data have identified poorer outcomes in B1 cases with left atrial enlargement [6]. Dogs in stage B2 represent a much more heterogeneous group of animals, with some individuals presenting a slow progression of the disease and others rapidly developing congestive heart failure (CHF) [7, 8]. Therefore, effective risk stratification in preclinical MMVD is crucial for accurate prognosis communication with owners and personalized clinical management strategies encompassing monitoring and treatment decisions.

Cardiac risk stratification in dogs with preclinical MMVD is a complex and composite assessment that should take into consideration clinical, electrocardiographic, radiographic, echocardiographic, and possibly biomarker findings in light of breed, sex, age, and possible comorbidities of each patient [9, 10, 11, 12, 13, 14, 15, 16, 17]. However, the development of truly predictive risk stratification schemes awaits further refinement, as stated in the most recent ACVIM consensus guidelines [4]. The Mitral INsufficiency Echocardiographic (MINE) score is an easy‐to‐use echocardiographic classification of the severity of MMVD, able to stratify the risk of cardiac and all‐cause mortality in dogs with MMVD [18].

The primary aim of this study was to verify the efficacy of the MINE score in stratifying the cardiac risk in a large sample of preclinical dogs with MMVD. Secondary aims were to propose a definition of “advanced B2” based on the MINE score, and to evaluate a possible simplification of the original MINE score, called “MINE score 2”.

## 2 Materials and Methods

This retrospective, multicenter, cohort study was conducted across eight European cardiologic referral centers including six in Italy, one in Norway, and one in the United Kingdom. The study reviewed data from privately owned dogs diagnosed with preclinical MMVD (inclusion criterion). The study subject recruitment period was related to the available database of each center, as follows: Anicura Istituto Veterinario Novara from January 2011 to December 2021, Anicura Clinica Veterinaria CMV Varese from June 2011 to December 2021, Clinica Veterinaria Milano Sud from June 2014 to December 2021, Department of Animal Medicine, Production and Health of the University of Padua from March 2014 to December 2021, the Department of Veterinary Sciences of the University of Pisa from April 2015 to December 2021, the HeartVets from December 2015 to December 2021, and the Anicura Oslo Animal Hospital from January 2016 to December 2021, the Department of Veterinary Medicine of the University of Perugia from August 2017 to December 2021.

Due to the retrospective study design, no institutional animal care and use approval or client consent was sought.

### 2.1 Animals

All dogs had to have undergone a complete physical examination, chest radiographs, and echocardiography on the same day to be included in the study. Data regarding breed, sex, age, body weight, ACVIM stage, echocardiographic variables, and baseline treatment were obtained from case records. Clinical ACVIM classification was verified and updated, when needed, to the most recent guidelines [4].

Dogs aged less than 3 years old were excluded to limit the possibility of inadvertently including congenital mitral valve disease. Moreover, dogs weighing more than 20 kg were excluded because MMVD has a different pathophysiology and prognosis in medium‐to‐large breed dogs. In addition, dogs having a fractional shortening of less than 25%, presenting atrial fibrillation, presenting other cardiac diseases or systemic diseases, receiving diuretics or other cardiovascular drugs apart from pimobendan, ACE‐inhibitors, and spironolactone were excluded.

### 2.2 Echocardiographic Examination and Scoring System

Transthoracic echocardiographic examinations had been carried out with ultrasound machines equipped with phased‐array transducers and a simultaneous single‐lead electrocardiogram. Dogs were imaged from right and left parasternal positions, and standard echocardiographic two‐dimensional, M‐mode, and Doppler images were acquired without sedation [19].

Based on the MINE score [18], four echocardiographic variables were considered: (i) left atrium‐to‐aorta ratio (LA/Ao), obtained from the right parasternal short‐axis view [20]; (ii) left ventricular end‐diastolic diameter normalized for body weight (LVIDDn), measured on the M‐mode obtained from the right parasternal short‐axis view [21]; (iii) left ventricular fractional shortening (FS), measured on the M‐mode obtained from the right parasternal short‐axis view [21]; and (iv) E‐wave transmitral peak velocity (E‐vel), obtained with the pulsed‐wave Doppler from the left apical 4‐chamber view [11].

### 2.3 Survival

Long‐term outcome and survival were assessed by reviewing clinical databases and by telephone interviews with the owners or the referring vets. For the aim of the study, a composite cardiac endpoint was considered, including both occurrence of CHF or cardiac death. The diagnosis of CHF was wherever possible supported by evidence from thoracic radiographs demonstrating cardiogenic pulmonary edema. If a dog's clinical condition precluded obtaining thoracic radiographs, a clinical diagnosis of CHF was made based on compatible clinical signs and physical examination that could not be explained by another disease based on clinical judgment and amelioration of clinical signs after diuretic treatment. The cause of death was recorded. Death was classified into cardiac‐related cause and non‐cardiac‐related cause. Cardiac death was defined as death secondary to cardiogenic pulmonary edema or euthanasia due to refractory CHF. All dogs that died unexpectedly with no other apparent cause for death were assumed to have experienced sudden cardiac death and were considered as cardiac‐related death. The survival data was finally updated in December 2022, and the statistical analysis was based on this final update.

### 2.4 Statistical Analysis

Descriptive statistics were generated. Numerical variables were reported as mean ± standard deviation or median (25th‒75th percentile) based on data distribution. The multivariable Cox proportional analysis (stepwise method) was used to evaluate the echocardiographic independent predictors of cardiac endpoint. Age, sex, and BW were considered as adjusting factors but not included in the model. Factors identified with the Cox proportional hazards analysis were tested for proportionality of hazards using Schoenfeld residual plots. The factors identified as independent predictors of outcome were then used to create scores, which were further classified into 4 categories of severity, as had been done for the first study. The differences in time to cardiac endpoint for each category of severity were then analyzed using Kaplan–Meier curves and the log‐rank test, with dogs suffering a non‐cardiac‐related death or still alive at the end of the study being censored. Dogs with complete lack of follow‐up information after first evaluation at each center were not considered for the statistical analysis. To assess the potential impact of this exclusion, we conducted a simple quantitative bias analysis simulating three extreme and intermediate scenarios regarding the outcome status of the excluded dogs: (1) all excluded dogs did not reach cardiac endpoint (no cardiac endpoint); (2) all excluded dogs reached the cardiac endpoint (worst‐case scenario); (3) 50% did not reach cardiac endpoint, 50% experienced the cardiac endpoint. The sensitivity analysis was used to assess the change in the overall event rate considering the different scenarios. Statistical analysis was performed with commercially available statistical software (GraphPad Prism, GraphPad Software Inc., San Diego, California; MedCalc Statistical Software version 22.006, MedCalc Software Ltd., Ostend, Belgium). A value of *p* < 0.05 was considered statistically significant.

## 3 Results

A total of 799 dogs were initially identified for inclusion in the study. Of these, 50 dogs (6.25%) were excluded due to complete lack of follow‐up data, resulting in a final study sample of 749 dogs with preclinical MMVD with follow‐up information. Of these, 304 were females and 445 were males, with a median age of 11 years (8.9–12.8 years) and a median body weight of 7.9 kg (5.3–10.2 kg). Most dogs were mixed‐breed (*n* = 219), followed by Cavalier King Charles Spaniel (*n* = 129), Chihuahua (*n* = 73), Dachshund (*n* = 50), Poodle (*n* = 41), Pinscher (*n* = 27), Jack Russel Terrier (*n* = 24), Yorkshire Terrier (*n* = 22), Pomeranian (*n* = 18), Shih‐tzu (17), Maltese (*n* = 15), Bichon (*n* = 13), Cocker Spaniel (*n* = 11), Beagle (*n* = 10), Pekingese (10), and the remaining 70 dogs were of 24 other breeds.

According to the ACVIM classification, 374 dogs were in stage B1 and 375 dogs in stage B2. Regarding treatment at the time of enrolment, defined as treatment after the first evaluation of each center, among B1 cases, 356 dogs (95%) did not receive any drug, 7 dogs (2%) received an ACE‐inhibitor in monotherapy, 4 dogs (1%) received an ACE‐inhibitor in association with pimobendan, 4 dogs (1%) received pimobendan in monotherapy, and 3 dogs (1%) received an ACE‐inhibitor in association with spironolactone. Among stage B2, 289 dogs (77%) received pimobendan monotherapy, 36 dogs (10%) received an ACE‐inhibitor in monotherapy, 19 dogs (5%) received pimobendan with an ACE‐inhibitor, 16 dogs (4%) received an ACE‐inhibitor with spironolactone, and 15 dogs (4%) received pimobendan with an ACE‐inhibitor and spironolactone.

In the study, 200 dogs (27%) reached the cardiac endpoint, and the rest of the sample (*n* = 549 dogs) did not reach the cardiac endpoint because they died of a non‐cardiac reason (*n* = 172 dogs) or were still alive at the last update in December 2022 (*n* = 377 dogs) with a median follow‐up period of 781 days (455–1281 days). This observed event rate of 27% (200/749 dogs) was used to quantify the possible selection bias of excluding the 50 dogs with complete lack of follow‐up information from the statistical analysis. When incorporating these 50 excluded dogs into the analysis under a possible scenario, the event rate varied as follows: (1) all no cardiac endpoint: 200/799 (25.0%); (2) all cardiac endpoint: 250/799 (31.3%); (3) 50% no cardiac endpoint and 50% cardiac endpoint: 225/799 (28.2%). These sensitivity analyses demonstrate that, even in the most extreme case, the overall event rate would only increase by 4.6 percentage points.

In the Cox proportional analysis, after adjusting for age, sex and BW, the LA/Ao, the LVIDDn and the E‐vel were significantly associated with the occurrence of the cardiac endpoint, while the FS% was not associated (Table 1). Thus, it means that each unitary increase of these three variables increases risk of reaching the cardiac endpoint almost 2‐fold for the LA/Ao, 6‐fold for LVIDDn and 3‐fold for E‐vel. Thus, a simplified version of the MINE score, termed “MINE score 2,” was developed, excluding FS%, maintaining previous cutoffs for the three remaining echocardiographic variables and preserving the 4 severity classes while redefining their reference intervals (Tables 2 and 3). We redefined the severity classes by examining the Kaplan–Meier curves for each numerical value of the MINE score 2, and identifying the points of separation of different curves (i.e., grouping those numerical values with similar time to cardiac endpoint).

**TABLE 1:** Results of the multivariate Cox proportional analysis (stepwise method). The covariates adjusted for age, sex and body weight that resulted associated with cardiac endpoint are reported in the table. Total number of subjects: 749 dogs. Number of outcome positive subjects: 200 dogs.

Covariate	*b*	SE	Wald	*p*	HR (95% CI)
LA/Ao	0.6018	0.2578	5.4509	0.0196	1.8 (1.1 to 3.0)
LVIDDn	1.8249	0.4099	19.8186	< 0.0001	6.2 (2.8 to 13.8)
E‐vel (m/s)	1.2321	0.3283	14.0878	< 0.0002	3.4 (1.8 to 6.5)

Abbreviations: *b*, regression coefficient; CI, confidence interval; HR, hazard ratio, SE, standard error.

**TABLE 2:** Selected echocardiographic cutoffs and relative scores of the simplified version of the MINE score (named “MINE score 2”).

	1	2	3	4
LA/Ao	< 1.70	1.70–1.90	1.91–2.50	> 2.50
LVIDDn	< 1.70	1.70–2.00	2.01–2.30	> 2.30
E‐vel (m/s)	< 1.20	1.20–1.50	> 1.50	

Abbreviations: E‐vel, E‐wave transmitral peak velocity; LA/Ao, left atrium‐to‐aorta ratio; LVIDDn, left ventricular end‐diastolic diameter normalized for body weight.

**TABLE 3:** Severity classification based on the total score obtained from the summation of the single scores obtained with Table 2.

Severity classification	Total score
Mild	3–4
Moderate	5–6
Severe	7–10
Late‐stage	11

**FIGURE 1:**
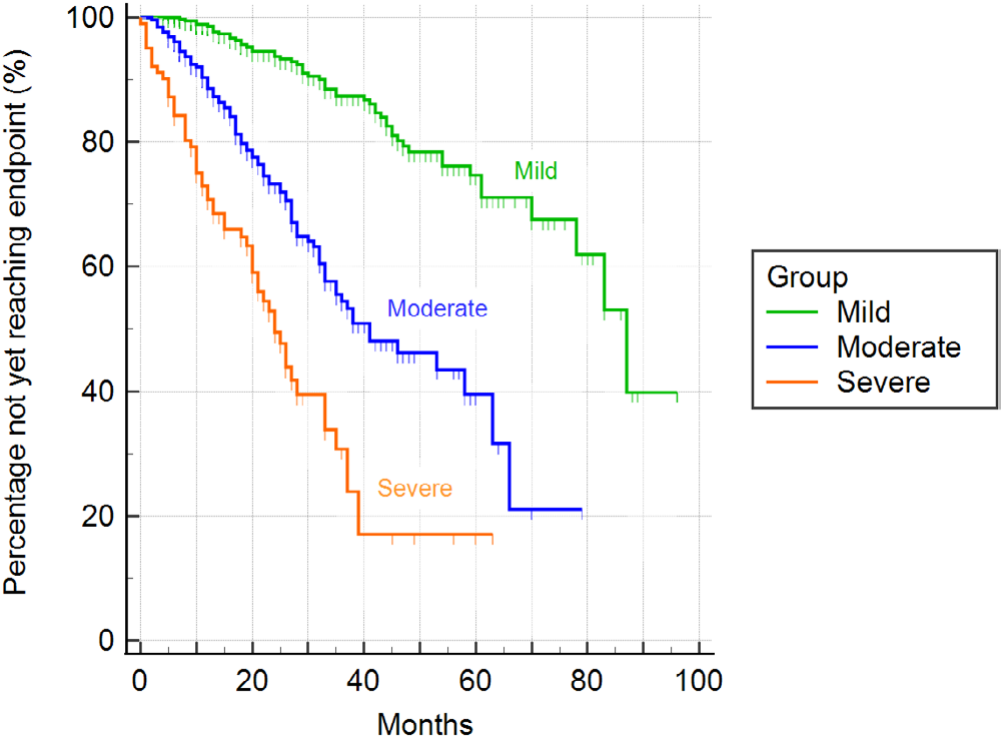
Kaplan–Meier curves illustrating the time to cardiac endpoint according to the severity classes of the MINE score 2 (*p* < 0.001).

**FIGURE 2:**
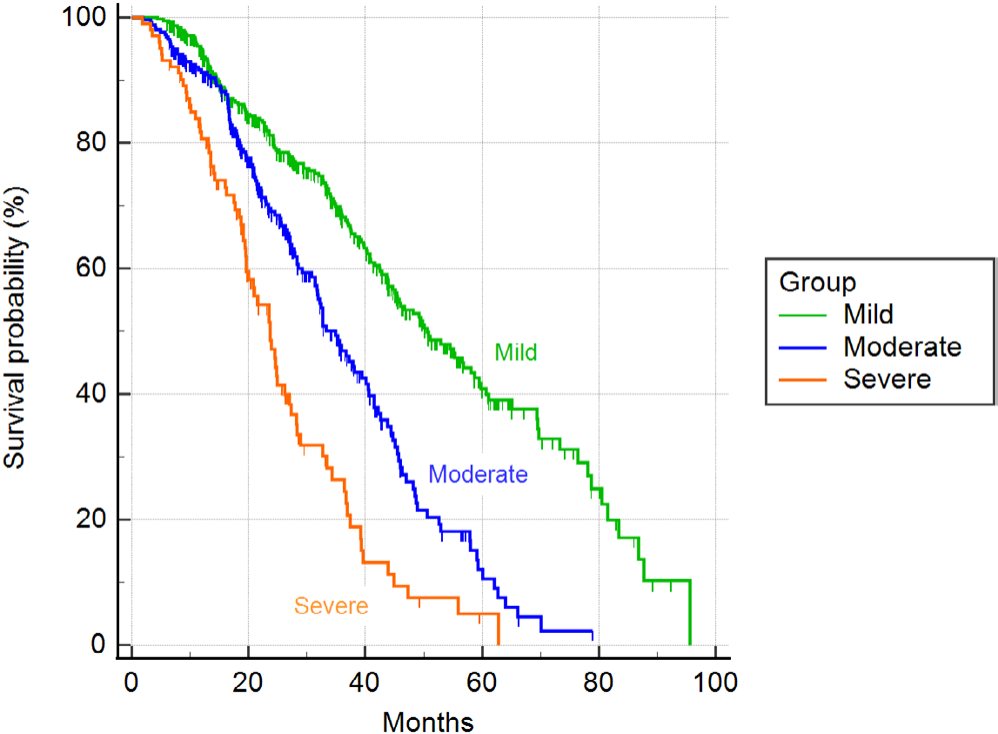
Kaplan–Meier curves illustrating survival time according to the severity classes of the MINE score 2, when all causes of death were considered (*p* < 0.001).

Based on the MINE score 2, 389 dogs (52%) were classified as mild, 258 (34%) as moderate and 102 (14%) as severe; no late‐stage cases were identified. Table 4 reported the baseline echocardiographic characteristics of the study sample. The median time to cardiac endpoint was statistically different between all the proposed severity classes: 2604 days for mild cases [95% confidence interval (CI) 2344–2604 days], 1216 days (95% CI: 998–1882) for moderate cases, and 718 days (95% CI: 599–980) for severe cases (chi‐square = 159.04; degrees of freedom (DF) = 2; *p* < 0.001; Figure 1). Statistically significant differences between all the proposed severity classes were present also considering all‐cause mortality: 1518 days for mild cases (95% CI 1353–1760 days), 1048 days (95% CI 943–1203 days) for moderate cases, and 710 days (95% CI 587–810 days) for severe cases (chi‐square = 94.52; DF = 2; *p* < 0.001; Figure 2).

**TABLE 4:** Baseline echocardiographic data of the study sample.

	Mild (*n* = 389)	Moderate (*n* = 258)	Severe (*n* = 102)
LA/Ao	1.44 (1.31–1.60)	1.84 (1.76–1.99)	2.10 (1.95–2.39)
LVIDDn	1.58 (1.45–1.69)	1.86 (1.77–1.94)	2.07 (1.99–2.19)
E‐Vel (m/s)	0.75 (0.64–0.86)	0.99 (0.88–1.11)	1.30 (1.20–1.41)

*Note:* Data are reported as median (25th–75th percentile).

Abbreviations: E‐vel, E‐wave transmitral peak velocity, LA/Ao, left atrium‐to‐aorta ratio; LVIDDn, left ventricular end‐diastolic diameter normalized for body weight.

**FIGURE 3:**
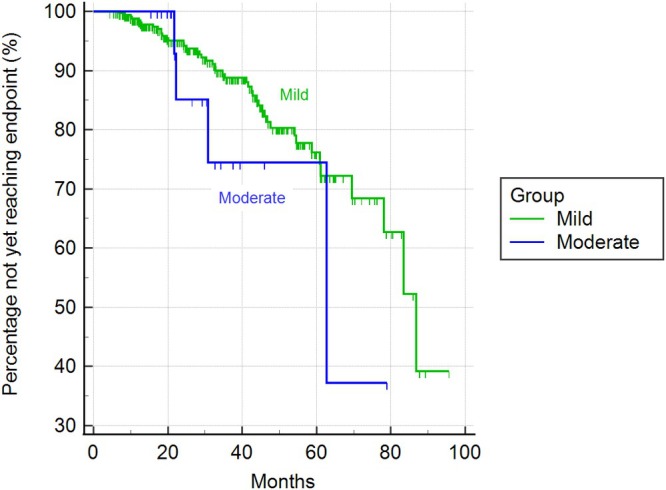
Kaplan–Meier curves illustrating the time to cardiac endpoint among dogs in stage B1 classified according to the MINE score 2 (*p* = 0.24).

**FIGURE 4:**
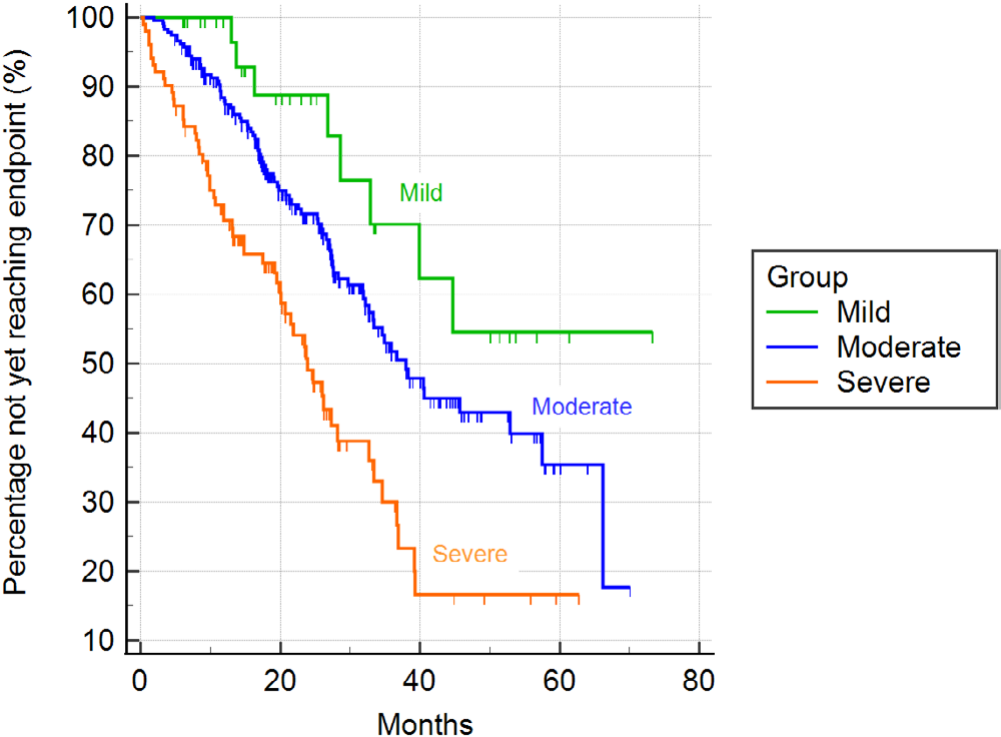
Kaplan–Meier curves illustrating the time to cardiac endpoint among dogs in stage B2 classified according to the MINE score 2 (*p* < 0.001).

Based on the ACVIM stage, dogs were classified into severity classes using the MINE score 2. Among dogs in stage B1, 352 (94%) were categorized as mild and 22 (6%) as moderate. In stage B2, 36 (10%) were deemed mild, 237 (63%) moderate, and 102 (27%) severe. There were no statistically significant differences in the median time to reach the cardiac endpoint between mild (2604 days; 95% CI 2344 to 2604 days) and moderate cases (1882 days; 95% CI 923 to 1882 days) within stage B1 (chi‐square = 1.39; DF = 1; *p* = 0.24; see Figure 3). In stage B2, severe cases exhibited a statistically shorter median time to reach the cardiac endpoint (718 days; 95% CI 599–980 days) compared to moderate cases (1141 days; 95% CI 980–1725 days) and mild cases (information not available; chi‐square = 26.01; DF = 2; *p* < 0.001; Figure 4).

## 4 Discussion

The current study supports the MINE score as an effective outcome‐based tool for stratifying cardiac risk in pre‐clinical MMVD. The original study [18] had already indicated the MINE score's efficacy in risk stratification at the preclinical stage; this study included a larger sample of preclinical dogs (749 vs. 398 cases) across a broader range of enrolling centers (eight centers versus two) across multiple countries. Furthermore, this study demonstrated that a simplified version of the original score using only three echocardiographic variables is effective. Fractional shortening (FS%) did not show independent association with cardiac outcomes in the current investigation. Previous studies have highlighted the prognostic significance of FS% in canine MMVD, indicating a progressive increase in FS% with worsening regurgitant fraction due to increased preload and reduced afterload [14, 22, 23, 24]. However, the FS% is strongly load‐dependent and influenced by sympathetic tone and may not consistently correlate with cardiac outcomes in MMVD [10, 17]. Consequently, FS% was not included in the computation of the MINE score 2. Based on three commonly utilized echocardiographic variables for left cardiac remodeling (LA/Ao and LVIDDn) and left ventricular filling pressure (E‐vel), MINE score 2 effectively stratifies cardiac risk in preclinical mitral valve disease, also providing valuable prognostic insights into all‐cause mortality. The severity of MMVD represents, in fact, a relevant comorbidity that could potentially impact the prognosis of concurrent conditions, serving as a presumptive marker for non‐cardiac diseases [25].

There are other proposals of MMVD severity scores; in 2015, López‐Alvarez et al. [26] proposed a clinical severity score system based on clinical signs and physical examination findings. In addition, other echocardiographic scoring systems were based on mitral valve leaflet anatomy, left atrial size, left ventricular size, color Doppler regurgitant jet area, spectral Doppler mitral inflow, and continuous wave Doppler regurgitant jet density scores [27, 28, 29]. However, many of these variables are based on a subjective assessment and the association of these scores with survival time was not evaluated. In addition, a mitral regurgitation severity index based on age, heart rate, LA/Ao or radiographic vertebral left atrial size has been recently described as a numerical tool for predicting outcomes in stage B2 dogs [30], nonetheless it does not aim to stratify the disease into severity classes.

Echocardiographic evaluation of MMVD severity in dogs mirrors approaches in human medicine [31, 32, 33, 34]. It relies on assessing left cardiac remodeling, quantifying mitral regurgitation through measures such as regurgitation jet size by color‐Doppler, effective regurgitant orifice area, proximal isovelocity surface area, vena contracta, and regurgitant fraction, as well as estimating left ventricular filling pressure using parameters like mitral inflow, isovolumetric relaxation time, pulmonary venous flow, regurgitant jet profile, and tissue Doppler echo variables [11, 14, 22, 23, 35, 36, 37, 38, 39, 40]. However, most of these techniques are time‐consuming and require multiple measurements. Furthermore, consideration must be given to intra‐ and inter‐observer variability, as these measurements typically demand skilled operator competence [41, 42, 43]. Consequently, we posit that the application of the MINE score 2 could offer an objective and easy assessment of dogs with MMVD with wide clinical generalizability, potentially influencing the clinical and therapeutic management of the disease. Future comparative studies exploring the prognostic value of the MINE score against more advanced metrics for estimating mitral regurgitation severity, such as vena contracta and regurgitant fraction/volume, are warranted.

Reasonably, the most useful application of MINE score 2 is the stratification of cardiac risk in stage B2, a heterogeneous class in terms of severity and prognosis. The term “advanced B2” is commonly used to refer to asymptomatic dogs at high risk of CHF. The elevation of left atrial filling pressures without overt clinical congestion is termed hemodynamic congestion and precedes clinical congestion [44]. Heart failure is ultimately a hemodynamic disorder, and its management must target hemodynamics [44]. The concept of preclinical congestion and hemodynamic congestion is used in human medicine, but there is no consensus on that in veterinary medicine. So, a proposal is to define “advanced B2” those preclinical dogs classified as “severe” according to the MINE score 2. This could help in the communication process with owners and colleagues in terms of prognosis in MMVD and support in the design of clinical trials to evaluate if severe B2 could benefit from supplementary treatment in addition to pimobendan.

Our retrospective cohort study should be interpreted in light of its limitations. First, as a retrospective longitudinal study, cardiac rechecks were not homogeneous in the follow‐up period of each case after inclusion, and the administration of cardiac medications varied within the sample, both in terms of type and duration of treatment before and after inclusion. Although the vast majority of B1 cases (95%) did not receive any medication in accordance with the guidelines, a small minority of B1 (5%) received ACE‐inhibitors, pimobendan, or spironolactone, reflecting the real canine general population. No study has demonstrated that treatment at this stage of MMVD alters clinical outcomes. Similarly, while most B2 cases (86%) were prescribed pimobendan upon enrollment, the remaining 14% of B2 cases received only an ACE‐inhibitor in monotherapy or in association with spironolactone because they were evaluated before the availability of the EPIC trial results. While such treatment could have influenced the median time to cardiac events in our B2 sample, our survival curve of B2 dogs is remarkably similar to that observed in the EPIC trial. Second, the initial exclusion of the 6% of dogs with complete lack of follow‐up information could potentially introduce selection bias, particularly if their outcomes differed systematically from those included. However, quantitative bias analysis was conducted, and the relatively small proportion of excluded cases is unlikely to substantially affect the validity or direction of the main study findings. Third, the specific date of CHF was not ascertainable for all dogs, resulting in cardiac death, rather than CHF, being considered as the cardiac endpoint in approximately 25% of the cases. Moreover, the outcome assessed by telephone interviews with owners and referring veterinarians was verified by having the referring veterinarian share clinical data with the investigators, allowing the investigators to best define the precise outcome of each dog (e.g., clinical, radiographic or hospitalization reports); however, these objective data were not always available, and thus the outcome definition was based on subjective interpretation of owners' description of events in some cases. Moreover, clinically significant systemic diseases were mainly excluded by complete history and physical examination. Hematochemical tests and abdominal ultrasound were not performed in all cases; thus, we cannot totally exclude subclinical comorbidities in some cases. Lastly, the study cohort was derived from referral centers, potentially limiting generalizability to first opinion practices or other healthcare facilities.

## 5 Conclusions

The simplified version of the score (“MINE score 2”) is clinically effective for risk‐stratification of preclinical MMVD. This outcome‐based scoring system could be a complementary tool to ACVIM classification, helping to identify asymptomatic dogs at increased risk of CHF or cardiac death, and dogs in stage B2 classified as “severe” by the MINE score 2 can be defined “advanced” B2 stage.

## Disclosure

Authors declare no off‐label use of antimicrobials.

## Ethics Statement

Authors declare no institutional animal care and use committee or other approval was needed. Authors declare human ethics approval was not needed.

## Conflicts of Interest

The authors declare no conflicts of interest.
